# STIM2β is a Ca^2+^ signaling modulator for the regulation of mitotic clonal expansion and *PPARG2* transcription in adipogenesis

**DOI:** 10.1111/febs.70118

**Published:** 2025-05-09

**Authors:** Su Ji Jeong, Bo‐Woong Sim, Sun‐Uk Kim, Chan Young Park

**Affiliations:** ^1^ Department of Biological Sciences Ulsan National Institute of Science and Technology Ulsan Korea; ^2^ Futuristic Animal Resource & Research Center (FARRC), Korea Research Institute of Bioscience and Biotechnology (KRIBB) Cheongju Korea; ^3^ Department of Biomedical Engineering Ulsan National Institute of Science and Technology Ulsan Korea

**Keywords:** adipogenesis, cell cycle regulation, intracellular Ca^2+^, PPARγ2, STIM2β

## Abstract

Intracellular Ca^2+^ is crucial in the regulation of adipocyte lipid metabolism and adipogenesis. In this study, we aimed to investigate the regulation mechanism of intracellular Ca^2+^ levels ([Ca^2+^]_i_) during adipocyte differentiation. We found that the expression of stromal interaction molecule 2 beta (STIM2β), which is the inhibitor of store‐operated Ca^2+^ entry (SOCE), is upregulated throughout the differentiation process. Evaluation of [Ca^2+^]_i_ in 3 T3‐L1 and primary stromal vascular fraction (SVF) cells revealed that the basal Ca^2+^ level is downregulated after differentiation. Knockout (KO) of *STIM2β* in 3T3‐L1 and primary SVF cells showed increased [Ca^2+^]_i_, indicating the involvement of STIM2β in the regulation of [Ca^2+^]_i_ during adipogenesis. We further evaluated the function of STIM2β‐mediated [Ca^2+^]_i_ in early and terminal differentiation of adipogenesis. Analysis of cell proliferation rate during mitotic clonal expansion (MCE) in wild‐type and *STIM2β* KO 3T3‐L1 cell lines revealed that a larger population of KO cells underwent G1 arrest, suggesting that reduced [Ca^2+^]_i_ by STIM2β induces MCE. Additionally, ablation of STIM2β increased differentiation efficiency, with more lipid accumulation and rapid transcriptional activation of adipogenic genes, especially proliferator‐activator receptor γ2 (*PPARG2*). We found that *PPARG2* transcription is regulated by store‐operated calcium entry (SOCE) downstream transcription factors, confirming that increased [Ca^2+^]_i_ by STIM2β ablation promotes *PPARG2* transcription during adipogenesis. Additionally, *STIM2β* KO mice showed hypertrophic adipose tissue development. Our data suggest that STIM2β‐mediated [Ca^2+^]_i_ plays a pivotal role in the regulation of mitotic clonal expansion and *PPARG2* gene activation and provides evidence that MCE is not a prerequisite process for terminal differentiation during adipogenesis.

## Graphical abstract

STIM2β acts as the intracellular Ca^2+^ regulator during adipogenesis. By inhibiting intracellular Ca^2+^ levels and SOCE, STIM2β promotes mitotic clonal expansion in the early stage of adipogenesis. In the terminal differentiation stage, increased SOCE activity enhances *PPARG2* gene transcription. STIM2β induces mitotic clonal expansion and delays the expression of PPARγ2 proteins to regulate the proper cell numbers to be differentiated and adjust the rate of adipogenesis.

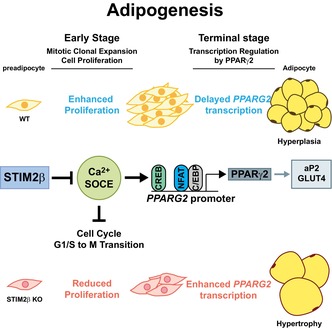

**Abbreviations**
AdipoQ: adiponectin
aP2: fatty acid binding protein 4
[Ca^2+^]_i_
: intracellular calcium levels
C/EBP: CCAAT/enhancer binding protein
CREB: cAMP‐responsive element binding protein
CsA: cyclosporine A
DD: differentiation day
Diff: differentiation
ER: endoplasmic reticulum
eWAT: epidydimal white adipose tissue
Gd^3+^
: gadolinium ion
GLUT4: glucose transporter 4
iWAT: inguinal white adipose tissue
KO: knockout
MCE: mitotic clonal expansion
NFAT: nuclear factor of activated T cells
PI: propidium iodide
PPARγ2: proliferator‐activator receptor gamma 2
qPCR: real‐time quantitative polymerase chain reaction
RT‐PCR: reverse transcription polymerase chain reaction
SOCE: store‐operated calcium entry
STIM: stromal Interacting Molecule
SVF: stromal vascular fraction
TG: thapsigargin
WT: wild‐type

## Introduction

Mature adipocytes play a pivotal role in energy homeostasis by efficiently storing excess energy. These cells dynamically adjust their size and lipid content in response to changing nutritional needs. Furthermore, adipose tissue functions as an endocrine organ by secreting adipokines that regulate biological processes such as food intake, insulin response, vascular remodeling, and immune responses. Consequently, adipocyte dysfunction contributes to metabolic diseases associated with obesity [1]. Therefore, efforts have been made to understand the mechanism of adipogenesis over the past several decades. In this quest, various *in vitro* cellular models, such as 3T3‐L1 pre‐adipocyte cells, have been used to establish the stages of adipogenesis: contact inhibition, mitotic clonal expansion (MCE), and terminal differentiation. Proliferated pre‐adipocytes become growth arrested by contact inhibition after reaching confluence. Upon hormonal induction, growth‐arrested cells re‐enter the cell cycle and undergo 2 to 4 rounds of replication; this stage is called MCE [2]. However, the requirement of MCE in adipogenesis is under debate [3, 4]. The terminal differentiation of adipogenesis is mediated by transcription factors, such as the members of the CCAAT/enhancer binding protein (C/EBP) family and the peroxisome proliferator‐activated receptor gamma (PPARγ) [5]. PPARγ exists in two isoforms, PPARγ1 and PPARγ2, resulting from different promoter usage and alternative splicing [6, 7]. PPARγ1 is ubiquitously expressed, while PPARγ2 is specifically expressed in adipocytes [8]. In particular, PPARγ2 plays a role in the transcriptional activation of adipocyte‐specific genes, such as aP2/fatty acid binding protein 4 (FABP4), Glucose Transporter 4 (GLUT4), and adiponectin (AdipoQ). Additionally, PPARγ2 induces the expression of C/EBPα, which acts as a coactivator of adipocyte‐specific genes. Depletion of PPARγ2 has been shown to reduce the differentiation of 3T3‐L1 cells [9] and restrict adipose tissue expansion in mice [10]. In contrast, overexpression of PPARγ2 induces adipocyte differentiation in fibroblasts [11]. Therefore, PPARγ2 is considered the master regulator of adipogenesis.

Adipogenesis is governed by a complex interplay of signaling pathways. As a secondary messenger, intracellular Ca^2+^ regulates cellular signal transduction during adipocyte differentiation [12, 13, 14, 15]. However, the regulation mechanism of [Ca^2+^]_i_ in adipogenesis is poorly understood. Several studies suggest that store‐operated Ca^2+^ entry (SOCE) plays a crucial role in regulating adipogenesis [16, 17, 18]. SOCE is the major process of Ca^2+^ influx across the plasma membrane (PM) in non‐excitable cells and is known to regulate Ca^2+^ homeostasis. SOCE is mediated by two key proteins, stromal interaction molecule (STIM) and Orai Ca^2+^ channel. STIM, an endoplasmic reticulum (ER) transmembrane protein with a Ca^2+^ sensing domain (EF‐hand), oligomerizes upon ER Ca^2+^ depletion. Subsequently, it is translocated to the ER‐PM junction and interacts with Orai channels located at the PM to trigger a Ca^2+^ influx [19, 20, 21]. This Ca^2+^ influx not only refills intracellular Ca^2+^ stores but also evokes various Ca^2+^‐response signaling cascades to control gene transcription, cell cycle, apoptosis, and other cellular processes [22].

SOCE is tightly regulated by diverse mechanisms [23]. One of the intriguing mechanisms is governed by STIM2β, an alternative spliced isoform of STIM2. Diverging from other STIM2 isoforms that activate Orai Ca^2+^ channels, STIM2β acts as the negative regulator of SOCE [24, 25]. The distinctive feature of STIM2β lies in the inclusion of an additional exon (STIM2 exon 9). This exon introduces eight amino acids into the CRAC activating domain (CAD) that is critical for the binding and activation of Orai1 and the stabilization of STIM oligomers. Consequently, STIM2β inhibits Orai channel activation through an allosteric mechanism by forming hetero‐oligomer with other STIM isoforms [26]. This unique feature of STIM2β as a negative regulator of SOCE highlights the possibility that SOCE activity could be regulated by alternative splicing of STIM2 during various cellular events. A previous study revealed that upregulated STIM2β regulates basal Ca^2+^ levels and Ca^2+^‐dependent transcription factors, thereby modulating myogenesis [27].

Here, we suggest that STIM2β is an unknown intracellular Ca^2+^ regulator during adipogenesis. We found that STIM2β modulates intracellular Ca^2+^ levels and SOCE activity during the differentiation of 3T3‐L1 pre‐adipocytes and primary stromal vascular fraction (SVF) cells. In addition, the changes in intracellular Ca^2+^ dynamics by STIM2β were involved in the regulation of the cellular proliferation rate during MCE. In the terminal stage of adipogenesis, the SOCE activity positively regulated the transcription of the PPARγ2 genes with Ca^2+^‐dependent transcription factors. Our findings highlight the importance of intracellular Ca^2+^ level regulation for the proper processing of each stage of adipogenesis, and STIM2β balances the cell proliferation and terminal differentiation process.

## Results

### STIM2β is upregulated and regulates [Ca^2+^]_i_ during Adipogenesis

Store‐operated Ca^2+^ entry (SOCE) is known to be involved in various cellular processes, including cellular differentiation. Stromal interacting molecule 2 beta (STIM2β) has distinctive properties compared to other STIM homologs and isoforms, as STIM2β inhibits SOCE while others activate SOCE. However, less is known about the physiological role of STIM2β, leaving room for further studies. Previously, we reported that Ca^2+^ signaling was regulated by STIM2β, a novel SOCE modulator, during myogenesis [27]. Given that both myoblasts and pre‐adipocytes originate from mesenchymal stem cell lineage, we also questioned whether STIM2β‐mediated Ca^2+^ signaling and SOCE are involved during adipogenesis.

**Fig. 1:**
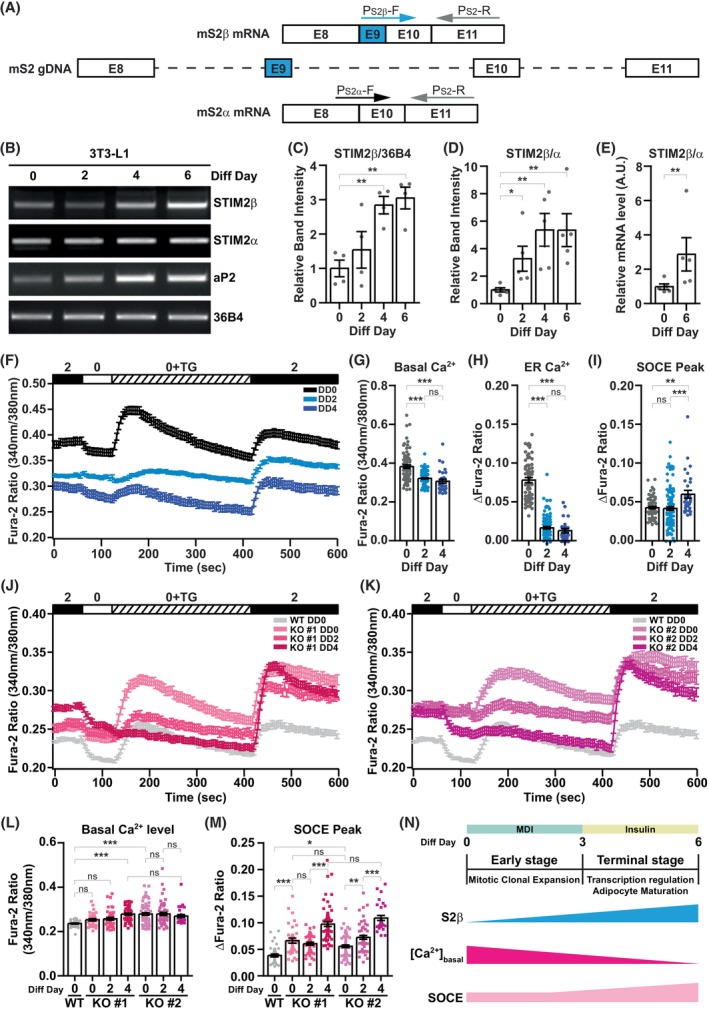
STIM2β is upregulated and decreases intracellular Ca^2+^ levels ([Ca^2+^]_i_) during differentiation of 3T3‐L1 pre‐adipocytes. (A) Schematic description of PCR primers specific for STIM2α and STIM2β. (B) Reverse transcription polymerase chain reaction (RT‐PCR) analysis of the mRNA expression of STIM2 alternative splicing variants in 3T3‐L1 cells at the indicated differentiation day (Diff Day). This experiment was repeated more than 5 times using independent sets of differentiated samples. (C) Bar graphs represent the RT‐PCR band intensity of STIM2β relative to 36B4; 4 independent gel images were used to analyze the band intensity of STIM2β. (D) Relative expression level of STIM2β mRNA relative to STIM2α in 3T3‐L1 cells. 5 different gel images were used to analyze the expression of STIM2β mRNA levels. An unpaired *t*‐test was used for the statistical evaluation of (C) and (D). (E) Quantitative real‐time polymerase chain reaction (qPCR) analysis of relative expression levels of STIM2 alternative splicing variants in 3T3‐L1 cells. 5 different sets of cDNA samples were used for the analysis. An unpaired *t*‐test was used to evaluate the statistical significance. (F) [Ca^2+^]_i_ analysis in undifferentiated (Differentiation Day 0; DD0) and differentiated (Differentiation Day 2; DD2 and Differentiation Day 4; DD4) 3T3‐L1 cells. [Ca^2+^]_i_ were expressed as ratios of 340:380 nm Fura‐2 fluorescence signals. (DD0, *n* = 77; DD2, *n* = 116; DD4, *n* = 30). (G) Bar graph presents the basal Ca^2+^ levels of cells in (F). Basal Ca^2+^ level was calculated by averaging Fura‐2 ratios of 340:380 nm fluorescence signals from 30 to 60 s. The one‐way ANOVA test combined with Bonferroni's Multiple Comparison Test was used to evaluate statistical significance. (H) Bar graph presents the ER Ca^2+^ release by thapsigargin (TG) treatment in (F). The ER Ca^2+^ release was calculated by subtracting the minimum Fura‐2 ratio from the maximum Fura‐2 ratio. These ratios were obtained by averaging the values from three imaging points preceding and following the respective maximum and minimum points. The one‐way ANOVA test combined with Bonferroni's Multiple Comparison Test was used to evaluate statistical significance. (I) Bar graph presents the store‐operated Ca^2+^ entry (SOCE) peak after 2 mm Ca^2+^ add‐back in (F). SOCE peak was calculated by subtracting the minimum Fura‐2 ratio from the maximum Fura‐2 ratio. These ratios were obtained by averaging the values from three imaging points preceding and following the respective maximum and minimum points. The one‐way ANOVA test combined with Bonferroni's Multiple Comparison Test was used to evaluate statistical significance. The data presented in (F–I) is representative, and the [Ca^2+^]_i_ analysis experiment was repeated more than 5 times. (J) [Ca^2+^]_i_ analysis in STIM2β knockout(KO) #1 3T3‐L1 cell line at differentiation day 0, 2, and 4 (presented as DD0, DD2, and DD4). Wild‐type (WT) 3T3‐L1 cells at differentiation day 0 (presented as WT DD0) were used as control (WT DD0, *n* = 39; KO #1 DD0, *n* = 35; KO #1 DD2, *n* = 40; KO #1 DD4, *n* = 59). (K) [Ca^2+^]_i_ analysis in STIM2β KO #2 3T3‐L1 cell line at differentiation day 0, 2, and 4 (presented as DD0, DD2, and DD4). WT 3T3‐L1 cells at differentiation day 0 (WT DD0) were used as control (WT DD0, *n* = 39; KO #1 DD0, *n* = 68; KO #1 DD2, *n* = 54; KO #1 DD4, *n* = 30). (L) Bar graph presents the basal Ca^2+^ levels of cells in (J) and (K). Basal Ca^2+^ level was calculated by averaging Fura‐2 ratios of 340:380 nm fluorescence signals from 30 to 60 s. The one‐way ANOVA test combined with Bonferroni's Multiple Comparison Test was used to evaluate statistical significance. (M) Bar graph presents the SOCE peak after 2 mm Ca^2+^ add‐back in (J) and (K). SOCE peak was calculated by subtracting the minimum Fura‐2 ratio from the maximum Fura‐2 ratio. These ratios were obtained by averaging the values from three imaging points preceding and following the respective maximum and minimum points. The one‐way ANOVA test combined with Bonferroni's Multiple Comparison Test was used to evaluate statistical significance. The data presented in (J–M) is representative, and the [Ca^2+^]_i_ analysis experiment was repeated more than 5 times. (N) Schematic description of changes in STIM2β expression levels and [Ca^2+^]_i_ during adipogenesis; Bars represent mean ± SEM. Levels of significance are as follows: ns, not significant; *P < 0.05; **P < 0.01; ***, *P* < 0.001.

To investigate whether STIM2β alternative splicing occurs during adipogenesis, we induced differentiation of 3T3‐L1 cells and measured the mRNA expression of each STIM2 isoform (STIM2β and STIM2α) by using STIM2 exon9 flanking primer (P_S2β_‐F) and STIM2 exon8‐exon10 flanking primer (P_S2α_‐F) for each isoform (Fig. 1A). Reverse transcriptional PCR (RT‐PCR) analysis using specific primers for each STIM2 isoform revealed that the mRNA expression of STIM2β increased over time as the cells differentiated (Fig. 1B,C).

Due to the unique insertion of 8 amino acids encoded by exon9 on the CRAC activation domain (CAD), STIM2 variants STIM2β and STIM2α have opposite effects on SOCE activation (Fig. 1A). Therefore, we analyzed the expression ratio of SOCE inhibitor (STIM2β) over SOCE activator (STIM2α) in differentiating 3T3‐L1 cells and found that the expression of SOCE inhibitor STIM2β increased during adipogenesis (Fig. 1D). Further analysis of the expression ratio of STIM2 isoforms by real‐time quantitative PCR (qPCR) confirmed that SOCE inhibitor STIM2β expression is upregulated after differentiation (Fig. 1E).

STIM2 is known to regulate the basal cytosolic and ER Ca^2+^ levels [28]. Therefore, we hypothesized that increased STIM2β after differentiation would regulate the intracellular Ca^2+^ levels ([Ca^2+^]_i_) during adipogenesis. To investigate whether the changes in [Ca^2+^]_i_ occur during adipogenesis, we measured [Ca^2+^]_i_ using the Fura‐2 fluorescent Ca^2+^ indicator at different stages of adipogenesis: differentiation day 0 (DD0), differentiation day 2 (DD2) for the early stage, and differentiation day 4 (DD4) for the terminal stage (Fig. 1F). Analysis of basal cytosolic Ca^2+^ levels revealed reduced basal Ca^2+^ levels after differentiation (Fig. 1G). In addition, the ER Ca^2+^ release after thapsigargin (TG) treatment was also decreased during adipogenesis (Fig. 1H). We also evaluated the SOCE activity at each differentiation time point and found that SOCE activity was increased about 1.4‐fold at the terminal stage of differentiation (Fig. 1I). This increase in SOCE activity might be due to the increase in STIM1 expression during adipogenesis [16]. The SOCE activity at the early stage of differentiation (DD2) was not enhanced even though STIM1 expression was increased. However, upon closer examination, it was observed that some cells exhibited either higher or lower SOCE activity compared to day 0. This variation can be attributed to the occurrence of mitotic clonal expansion (MCE) during the early stage of differentiation. It is well known that SOCE activity varies at different stages of the cell cycle, with notable inhibition during the M phase [29, 30].

Analysis of [Ca^2+^]_i_ of 3T3‐L1 cells at different stages of adipogenesis revealed dynamic changes in basal cytosolic Ca^2+^ levels, ER Ca^2+^ contents, and SOCE activity. To investigate how STIM2β is involved in such dynamic changes in [Ca^2+^]_i_ during adipogenesis, we altered STIM2β expression in 3T3‐L1 cell lines by knockout (KO) and evaluated [Ca^2+^]_i_ changes after differentiation (Fig. 1J,K). We generated STIM2β KO 3T3‐L1 cell lines by targeting exon 9 with the CRISPR/Cas9 system. Two STIM2β KO 3T3‐L1 cell lines, with deletion of exon 9 in all 4 alleles, were selected for further examinations (Fig. S1A). The KO efficiency was confirmed by RT‐PCR, with no detectable STIM2β mRNA expression (Fig. S1B). In addition, STIM2β KO 3T3‐L1 cell lines did not show any increase in STIM2β mRNA expression upon differentiation, as confirmed by qPCR (Fig. S1C). The decrease in ER Ca^2+^ release by TG treatment was also observed in the STIM2β KO 3T3‐L1 cell lines. Notably, we could not observe any decrease in the basal cytosolic Ca^2+^ levels of STIM2β KO 3T3‐L1 cell lines after differentiation induction, confirming that the decreased basal Ca^2+^ levels were regulated by STIM2β (Fig. 1L). In addition, the SOCE activity at differentiation day 0 of STIM2β KO 3T3‐L1 cell lines was significantly higher than WT 3T3‐L1 cells, confirming the inhibitory effect of STIM2β on SOCE. STIM2β KO significantly enhanced the SOCE activity at the terminal stage of differentiation (DD4), with about a 2.53‐fold increase for the KO #1 cell line, and a 2.83‐fold increase for the KO #2 cell line in comparison to WT day 0 (Fig. 1M). Considering the 1.4‐fold increase in SOCE activity of WT 3T3‐L1 cells at differentiation day 4 (Fig. 1I), the increased STIM2β expression in WT 3T3‐L1 cells serves as a buffering mechanism for the SOCE activity increased by STIM1. We also found that there were no STIM2β KO cells with decreased SOCE activity in the early stage (DD2). Taken together, our data revealed that STIM2 alternative splicing occurs during adipogenesis to increase the expression of STIM2β. In addition, the increase in STIM2β expression resulted in the reduction of basal Ca^2+^ levels and SOCE activity, especially in the early stage of differentiation (Fig. 1N).

**Fig. 2:**
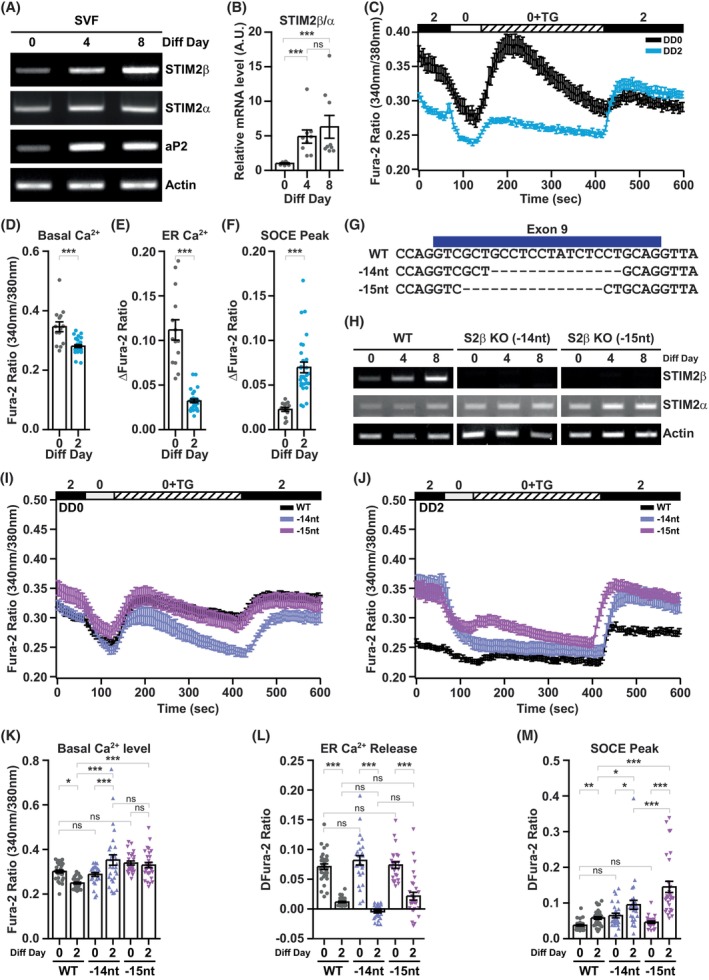
STIM2β is upregulated and decreases intracellular Ca^2+^ levels ([Ca^2+^]_i_) during the differentiation of primary stromal vascular fraction (SVF) cells. (A) mRNA expression analysis of STIM2 alternative splicing variants in primary SVF cells by RT‐PCR. This data is representative, and the experiment was repeated a total of 3 times. (B) Bar graphs represent the relative expression levels of STIM2β over STIM2α analyzed by qPCR. This data represents the combined results from 3 distinct cDNA sets, each analyzed in triplicate. Unpaired *t*‐test was used for the statistical evaluation. (C) [Ca^2+^]_i_ analysis of wild‐type (WT) SVF cells at differentiation day 0 (DD0) and differentiation day 2 (DD2). [Ca^2+^]_i_ were expressed as ratios of 340 : 380 nm Fura‐2 fluorescence signals. (DD0, *n* = 14; DD2, *n* = 29). (D) Bar graph presents the basal Ca^2+^ levels of cells in (C). Basal Ca^2+^ level was calculated by averaging Fura‐2 ratios of 340:380 nm fluorescence signals from 30 to 60 s; Unpaired *t*‐test was used for the statistical evaluation. (E) Bar graph presents the endoplasmic reticulum (ER) Ca^2+^ release by thapsigargin (TG) treatment in (C). The ER Ca^2+^ release was calculated by subtracting the minimum Fura‐2 ratio from the maximum Fura‐2 ratio. These ratios were obtained by averaging the values from three imaging points preceding and following the respective maximum and minimum points. Unpaired *t*‐test was used for the statistical evaluation. (F) Bar graph presents the store‐operated Ca^2+^ entry (SOCE) peak after 2 mm Ca^2+^ add‐back in (F). SOCE peak was calculated by subtracting the minimum Fura‐2 ratio from the maximum Fura‐2 ratio. These ratios were obtained by averaging the values from three imaging points preceding and following the respective maximum and minimum points; Unpaired *t*‐test was used for the statistical evaluation. The data presented in (C–F) is representative, and the experiment was repeated 3 times. (G) Sequence information of STIM2β exon 9 region of WT and STIM2β knockout (KO) mice. (H) mRNA expression of STIM2α and STIM2β in WT and STIM2β KO primary SVF cells after differentiation using RT‐PCR. This data is representative, and the experiment was repeated more than 5 times using independent sets of samples. (I) [Ca^2+^]_i_ analysis of WT and STIM2β KO SVF cells at differentiation day 0 (DD0). [Ca^2+^]_i_ were expressed as ratios of 340:380 nm Fura‐2 fluorescence signals. (WT, *n* = 43; −14 nt, *n* = 22; −15 nt, *n* = 25). (J) [Ca^2+^]_i_ analysis of WT and STIM2β KO SVF cells at differentiation day 2 (DD2). [Ca^2+^]_i_ were expressed as ratios of 340:380 nm Fura‐2 fluorescence signals. (WT, *n* = 62; −14 nt, *n* = 28; −15 nt, *n* = 30). (K) Bar graph presents the basal Ca^2+^ levels of cells in (I) and (J). Basal Ca^2+^ level was calculated by averaging Fura‐2 ratios of 340:380 nm fluorescence signals from 30 to 60 s. The one‐way ANOVA test combined with Bonferroni's Multiple Comparison Test was used to evaluate statistical significance. (L) Bar graph presents the ER Ca^2+^ release by TG treatment in (I) and (J). The ER Ca^2+^ release was calculated by subtracting the minimum Fura‐2 ratio from the maximum Fura‐2 ratio. These ratios were obtained by averaging the values from three imaging points preceding and following the respective maximum and minimum points. The one‐way ANOVA test combined with Bonferroni's Multiple Comparison Test was used to evaluate statistical significance. (M) Bar graph presents the SOCE peak after 2 mm Ca^2+^ add‐back in (I) and (J). SOCE peak was calculated by subtracting the minimum Fura‐2 ratio from the maximum Fura‐2 ratio. These ratios were obtained by averaging the values from three imaging points preceding and following the respective maximum and minimum points. The one‐way ANOVA test combined with Bonferroni's Multiple Comparison Test was used to evaluate statistical significance. The data presented in (I–M) is representative, and the experiment was repeated 3 times. Bars represent mean ± SEM. Levels of significance are as follows: ns, not significant; *, *P* < 0.05; **, *P* < 0.01; ***, *P* < 0.001.

To investigate whether this increase in STIM2β is a common occurrence in adipogenesis or a 3T3‐L1 cell line‐specific event, we further examined the changes in STIM2β expression in primary stromal vascular fraction (SVF) cells. We found that the mRNA expression of STIM2β in differentiated primary SVF cells is also increased over time by RT‐PCR (Fig. 2A). In addition, we measured the expression ratio of STIM2β and STIM2α by qPCR and confirmed that STIM2β alternative splicing is upregulated after the differentiation of SVF cells (Fig. 2B). We confirmed that an elevation in STIM2β expression level was consistently exhibited during adipogenesis with two different cell models.

We further examined the change in [Ca^2+^]_i_ during the differentiation of SVF cells using the Fura‐2 fluorescent Ca^2+^ indicator (Fig. 2C). As observed in 3T3‐L1 cells, the basal cytosolic Ca^2+^ levels of primary SVF cells were decreased at differentiation day 2 (Fig. 2D). In addition, the decrease in ER Ca^2+^ content was also observed in primary SVF cells (Fig. 2E). Interestingly, the SOCE activity of primary SVF cells was significantly enhanced at differentiation day 2 (Fig. 2F). The difference between 3T3‐L1 cells and primary SVF cells might be due to the cellular characteristics. 3T3‐L1 cells undergo mitotic clonal expansion during the early stage of differentiation, while the primary SVF cells do not proliferate once they reach confluency. Therefore, the primary SVF cells exhibit similar intracellular Ca^2+^ dynamics at the terminal differentiation stage of 3T3‐L1 cells (Fig. 1F).

To further confirm the role of STIM2β on the regulation of [Ca^2+^]_i_ during adipogenesis, we obtained primary SVF cells from WT (C57BL/6N) and STIM2β KO (C57BL/6N‐stim2β^em1^) mice for subsequent experiments. Briefly, STIM2β KO mice were generated using the CRISPR/Cas9 system. By microinjecting sgRNAs targeting STIM2 exon9 and *Cas9* mRNA into fertilized embryos of C57BL/6N mice, STIM2β founder mice were obtained. Founder mice were mated with WT C57BL/6N mice to separate alleles into heterozygote mice, which were further mated to generate homozygote mice. STIM2β exon9 deletion in homozygote mice was confirmed by Sanger sequencing, and we obtained 2 different deletion mutants (−14 nt and −15 nt) (Fig. 2G). Successful KO of STIM2β was confirmed by no detectable mRNA expression in RT‐PCR analysis. Furthermore, the increase in STIM2β transcription observed during adipogenesis was completely absent in STIM2β KO SVF cells (Fig. 2H).

We analyzed the [Ca^2+^]_i_ of WT and STIM2β KO SVF cell lines at differentiation day 0 (Fig. 2I) and differentiation day 2 (Fig. 2J). The basal cytosolic Ca^2+^ levels of WT and STIM2β KO primary SVF cells were not significantly different at differentiation day 0 but had slightly higher basal Ca^2+^ levels in −15 nt. The decrease in basal Ca^2+^ levels was not observed in STIM2β KO SVF cells at differentiation day 2. Interestingly, the basal Ca^2+^ level of −14 nt SVF cells was even upregulated after differentiation induction (Fig. 2K). The ER Ca^2+^ release after TG treatment was also analyzed in WT and STIM2β KO SVF cells. As observed in 3T3‐L1 cells, differentiation induction significantly reduced the ER Ca^2+^ levels, and there was no difference between WT and STIM2β KO SVF cells (Fig. 2L). In concordance with the previous data from 3T3‐L1 cell lines, the ablation of STIM2β only affected the basal cytosolic Ca^2+^ level change after differentiation. Next, we analyzed the SOCE activity in WT and STIM2β KO SVF cells. At differentiation day 0, the SOCE activity of both −14 nt and −15 nt was slightly but not significantly higher than that of WT cells. On differentiation day 2, the SOCE activity of WT SVF cells was increased by about 1.94‐fold in comparison to differentiation day 0. The SOCE activity of ‐14 nt SVF cells was higher by 2.89‐fold, and 4.38‐fold higher in ‐15 nt SVF cells in comparison to the SOCE activity of WT cells at differentiation day 0 (Fig. 2M). These data suggest that increased STIM2β in primary SVF cells regulates the SOCE activity during adipogenesis.

Taken together, we found that STIM2β alternative splicing is upregulated and regulates the intracellular Ca^2+^ dynamics during the differentiation of both 3T3‐L1 pre‐adipocytes and primary SVF cells.

### STIM2β regulates mitotic clonal expansion in the early differentiation stage

Analysis of intracellular Ca^2+^ dynamics during differentiation of 3T3‐L1 and primary SVF cells revealed that the SOCE activity in proliferating 3T3‐L1 cells during the early stage of differentiation shows distinctive features: cooccurrence of enhanced and reduced SOCE activity (Fig. 1I). In addition, the ablation of STIM2β in 3T3‐L1 cells vanished the feature (Fig. 1M). It is well known that intracellular Ca^2+^ signaling and SOCE regulate the cell cycle [31]. In addition, STIM2β‐mediated basal Ca^2+^ levels have been shown to directly regulate C2C12 myoblast proliferation [27]. Therefore, we hypothesized that the change in [Ca^2+^]_i_ mediated by STIM2β may regulate the mitotic clonal expansion of 3T3‐L1 cells during the early stage of adipogenesis.

**Fig. 3:**
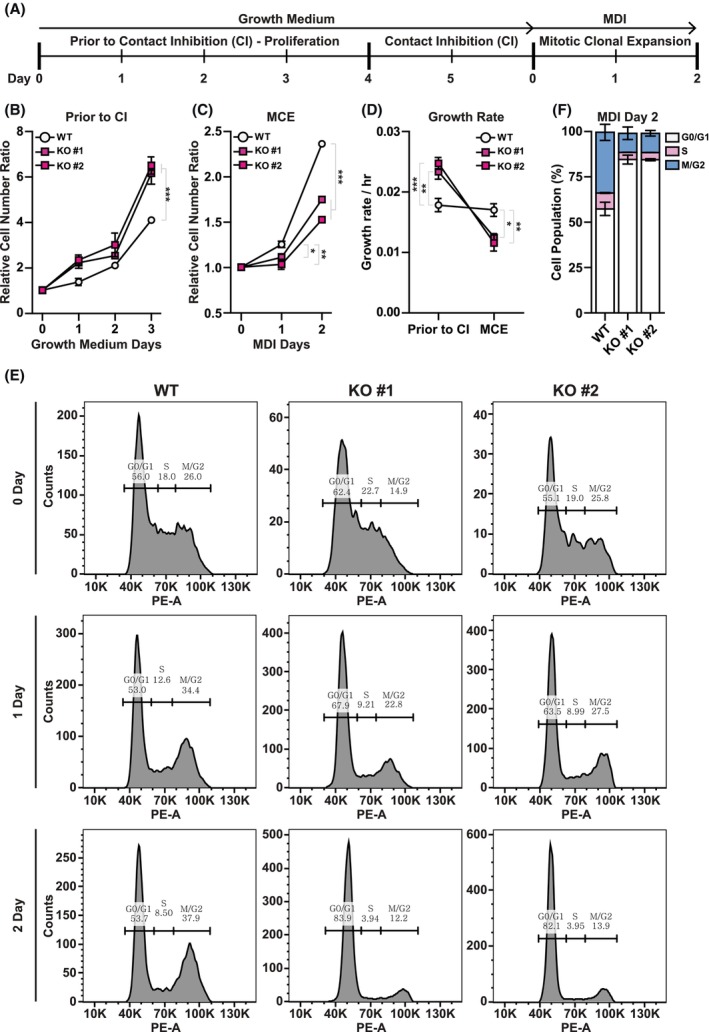
STIM2β‐mediated Mitotic Clonal Expansion (MCE) regulation during the early stage of adipogenesis. (A) Schematic description of proliferation status of 3T3‐L1 cells during adipogenesis. (B, C) Cell cycle analysis of 3T3‐L1 cells during adipogenesis. Comparison of increase in cell number between wild‐type (WT) and STIM2β knockout (KO) 3T3‐L1 cell lines in the growth medium (B) and differentiation medium (C). The cell number ratio was calculated at a given time point divided by the initial cell number of WT 3T3‐L1 cells. This data represents the combined results from 3 repeated experiments. (D) Comparison of growth rate before and after differentiation induction in WT and STIM2β KO 3T3‐L1 cell lines. The growth rate was calculated using cell numbers from (B) and (C). (E) Flow cytometric cell cycle analysis of WT and STIM2β KO 3T3‐L1 cell lines by PI staining. This data is representative, and the experiment was repeated 3 times. (F) The graph represents the percentage of the cells in each phase of the cell cycle at differentiation day 2. This data represents the combined results from 3 repeated flow cytometric cell cycle analyses using PI staining. Statistical significances are as follows: G0/G1 phase, WT vs KO #1: ***, WT vs KO #2: ***, KO #1 vs KO #2: ns; M/G2 phase, WT vs KO #1: **, WT vs KO #2: **, KO #1 vs KO #2: ns; S phase, WT vs KO #1: ns, WT vs KO #2: ns, KO #1 vs KO #2: ns; Two‐way ANOVA test combined with Bonferroni correction was used to evaluate the statistical significance. Levels of significance are as follows: ns, not significant; *, *P* < 0.05; **, *P* < 0.01; ***, *P* < 0.001.

To investigate whether the cell cycle regulation during adipogenesis is disrupted by STIM2β alteration, we measured the proliferation rate of WT and STIM2β KO 3T3‐L1 cell lines by counting cell numbers. WT and STIM2β KO L1 cells were seeded with the same density, and the cell numbers were counted at intervals of 24 h using a hemocytometer. We measured the cell numbers before contact inhibition states and after MDI treatment (Fig. 3A). Before contact inhibition (CI), in the growth medium condition, the proliferation rate of STIM2β KO 3T3‐L1 cells was higher than that of WT cells (Fig. 3B). However, after 2 days of contact inhibition followed by treatment of MDI, the proliferation rate of STIM2β KO 3T3‐L1 cell lines decreased in comparison to that of WT cells (Fig. 3C). We further analyzed the growth rate of each cell line at each stage of differentiation and found that the ablation of STIM2β reversed the growth rate after differentiation (Fig. 3D). These data suggest that increased basal Ca^2+^ levels and SOCE activity in STIM2β KO 3T3‐L1 cell lines inhibit the progression of mitotic clonal expansion (MCE).

SOCE activity throughout the cell cycle has been shown to vary across different phases of the cell cycle: a slight enhancement of SOCE during the G1 and S phases, and dramatic downregulation during the M phase [30, 32]. To investigate the effect of STIM2β‐mediated intracellular Ca^2+^ regulation on MCE more precisely, we further analyzed the cell cycle phases of WT and STIM2β KO 3T3‐L1 cell lines by propidium iodide (PI) staining (Fig. 3E). Interestingly, the M/G2 population of WT 3T3‐L1 cells was gradually increased during 2 days of differentiation. However, the M/G2 population in STIM2β KO 3T3‐L1 cell lines was not increased (Fig. 3E). We observed that almost 90% of cells showed cell cycle arrest at the G1/G0 phase in STIM2β KO 3T3‐L1 cell lines at differentiation day 2 (Fig. 3F).

Analysis of the cell cycle in the early stage of adipogenesis revealed that STIM2β deficiency inhibits MCE. These data suggest that STIM2β regulates MCE, probably by reducing the basal Ca^2+^ levels and inhibiting the SOCE to progress M/G2 phase.

### STIM2β inhibits terminal differentiation of adipogenesis

**Fig. 4:**
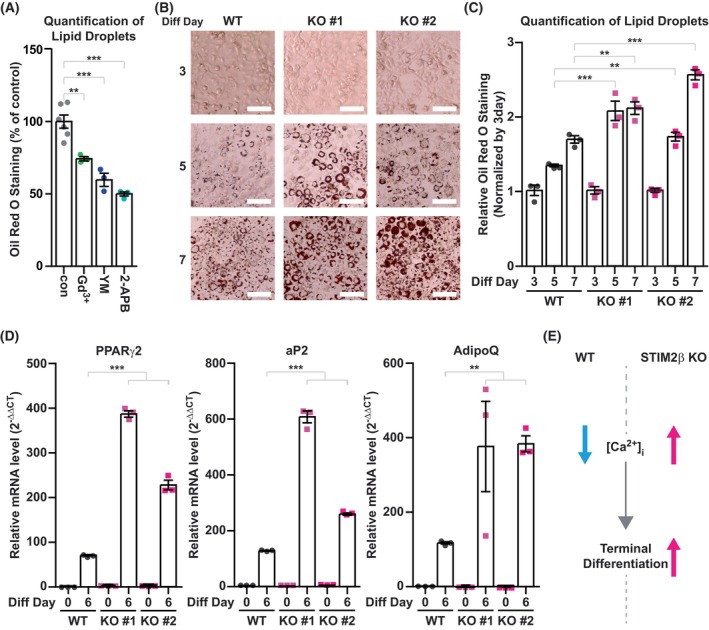
STIM2β deficiency enhances adipogenesis of 3T3‐L1 pre‐adipocytes. (A) Analysis of lipid accumulation in wild‐type (WT) 3T3‐L1 cells after treatment of store‐operated Ca^2+^ entry (SOCE) inhibitors. Cells were treated with 1 μm Gd^3+^, 1 μm YM58483, and 50 μm 2‐APB for 5 days with the differentiation medium. Differentiation medium‐only treated condition was used as a control to normalize the total amount of lipid quantified by Oil Red O staining. One‐way ANOVA test combined with Dunnett's Multiple Comparison test was used to evaluate statistical significance. This data is the combined results from 3 distinct sets of experiments. (B) Lipid accumulation and morphological change analysis by Oil Red O staining at the indicated time after hormonal differentiation induction of WT and STIM2β knockout (KO) 3T3‐L1 cell lines. Scale bars represent 100 μm; this data is representative, and the experiment was repeated 3 times. (C) Total lipid amount of (B) was analyzed by measuring the absorbance of Oil Red O at different time points of differentiation. The absorbance value of each time point was normalized by that on day 3 for each cell line. This data is the combined result from 3 distinct sets of experiments. A two‐way ANOVA test with Bonferroni correction was used to evaluate statistical significance. (D) Expression levels of adipogenic marker genes peroxisome proliferator‐activated receptor gamma 2 (PPARγ2), fatty acid binding protein 4 (aP2), and adiponectin (AdipoQ) were analyzed by qPCR to compare the efficiency of adipogenesis between WT and STIM2β KO 3T3‐L1 cell lines. This data represents triplicated results, and the experiment was repeated more than 3 times. A two‐way ANOVA test with Bonferroni correction was used to evaluate statistical significance. (E) Schematic description of the effect of STIM2β KO on intracellular Ca^2+^ levels and the terminal differentiation. Bars represent mean ± SEM. Levels of significance are as follows: **, *P* < 0.01; ***, *P* < 0.001.

Previous studies have shown that modification of [Ca^2+^]_i_ during adipogenesis can affect the differentiation efficiency [12, 13, 15, 33, 34]. To investigate whether SOCE is involved in the regulation of adipogenesis, we tested the effect of various orai Ca^2+^ channel blockers on the differentiation of 3T3‐L1 cells. We induced differentiation of 3T3‐L1 cells with or without orai1 blockers for 5 days of differentiation and analyzed the lipid droplets accumulation by oil red o staining. Treatment of orai1 blockers (1 μm of Gd^3+^, 1 μm of YM85483, and 50 μm of 2‐APB) successfully inhibited the SOCE induced by ER‐store depletion (Supplementary Fig. S2A,B). To ensure that SOCE is inhibited by orai1 blockers throughout the differentiation, we also measured the SOCE activity of cells that were treated with orai1 blockers for 5 days with MDI (Supplementary Fig. S2D,E). The negative SOCE peak values might have resulted from the photobleaching effect during live imaging, but clearly show that SOCE is successfully inhibited by inhibitors. After blocking orai1 activity with 1 μm of Gd^3+^, 1 μm of YM85483, and 50 μm of 2‐APB, the lipid droplet accumulation in 3T3‐L1 cells was significantly reduced (Fig. 4A). In addition, the basal cytosolic Ca^2+^ levels of 3T3‐L1 cells were significantly reduced after treatment with orai1 blockers (Supplementary Fig. S2C,F). These data further confirm that the decrease in the basal Ca^2+^ levels during adipogenesis was the result of STIM2β‐mediated inhibition of SOCE.

Decreased differentiation efficiency after blocking SOCE suggests that intracellular Ca^2+^ acts as the positive regulator of terminal differentiation of adipogenesis. To further investigate the role of STIM2β in terminal adipocyte differentiation, we compared the differentiation efficiency of WT and STIM2β KO 3T3‐L1 cell lines by inducing the differentiation with a hormonal cocktail. We first measured the lipid droplet accumulation of WT and STIM2β KO 3T3‐L1 cells by Oil Red O staining (Fig. 4B). On day 3 of differentiation, no significant difference was observed between WT and STIM2β KO 3T3‐L1 cell lines. However, on day 5 and day 7 of differentiation, more lipid droplets were observed in STIM2β KO cell lines (Fig. 4B). Furthermore, the amount of total lipid accumulation was significantly increased in both STIM2β KO cell lines (Fig. 4C), suggesting that STIM2β could play a role in modulating adipogenesis. Consistent with the observed phenotype, analysis of mRNA expression levels of adipogenic marker genes such as aP2, Adiponectin (AdipoQ), and PPARγ2 was significantly increased in STIM2β KO 3T3‐L1 cells compared to WT cells (Fig. 4D).

Taken together, our data suggest that SOCE is involved in the regulation of terminal differentiation of adipogenesis, and STIM2β functions as the negative regulator of adipogenesis by regulating the [Ca^2+^]_i_ during the differentiation process (Fig. 4E).

### Transcription of *PPARG2* is dependent on SOCE

**Fig. 5:**
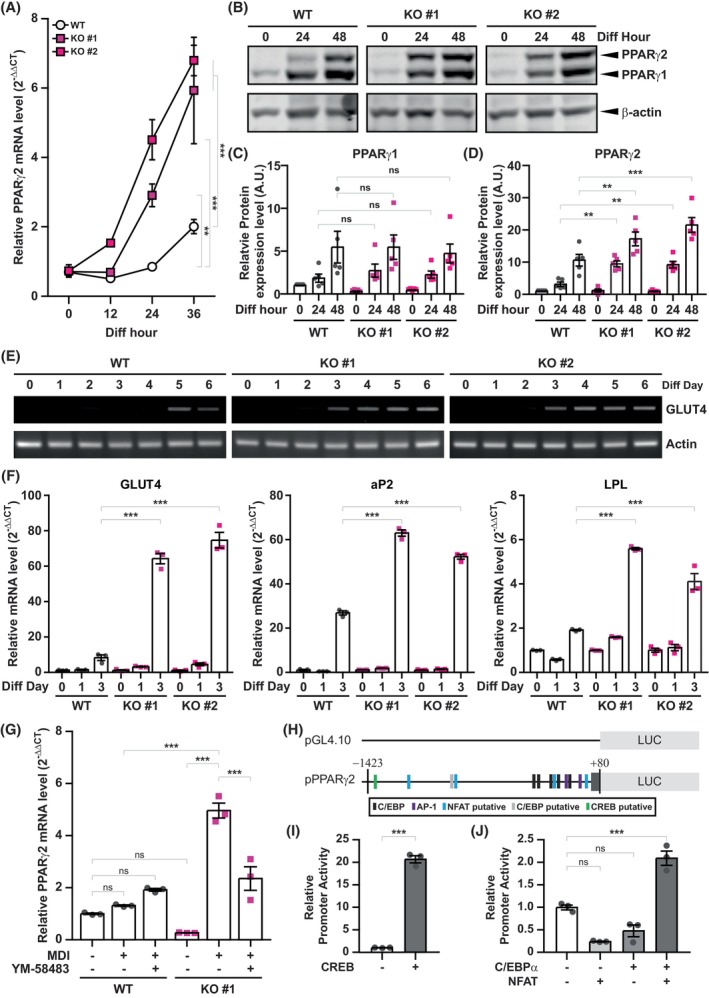
Increased peroxisome proliferator‐activated receptor gamma 2 (PPARγ2) transcription and activity in STIM2β knockout (KO) 3T3‐L1 cell lines. (A) qPCR analysis to compare the expression level of PPARγ2 between wild‐type (WT) and STIM2β KO 3T3‐L1 cell lines during early time points of differentiation. This data represents triplicated results, and the experiment was repeated more than 3 times. A two‐way ANOVA test combined with Bonferroni correction was used to evaluate the statistical significance. (B) Expression level of PPARγ isoforms after differentiation induction analyzed using western blotting. This data is representative, and this experiment was repeated for 4 times. (C, D) Protein expression in (B) was analyzed by measuring the band intensity ratio of PPARγ1 (C) and PPARγ2 (D) to β‐actin at each time point; 4 independent blots were used for the analysis. (E) Expression level of PPARγ2 downstream target gene glucose transporter 4 (GLUT4) analyzed using RT‐PCR. This data is representative and this experiment was repeated 3 times. (F) qPCR analysis of PPARγ2 downstream target genes glucose transporter 4 (GLUT4), fatty acid binding protein 4 (aP2), and lipoprotein lipase (LPL) at the indicated differentiation days (Diff Day). This data represents triplicated results, and the experiment was repeated more than 3 times. For the statistical analysis, a two‐way ANOVA test combined with Bonferroni correction was used. (G) Expression of PPARγ2 after store‐operated Ca^2+^ entry (SOCE) inhibitor (YM‐585483) treatment in WT and STIM2β KO#1 cells. Cells were treated with MDI with or without 1 μm YM‐58483 for 12 h. This data represents triplicated results, and the experiment was repeated 2 times. One‐way ANOVA test combined with Turkey's multiple comparison test was used to evaluate the statistical significance. (H) Graphical description of the PPARγ2 promoter–reporter system. (I, J) PPARγ2 promoter assay in HEK293 cells. This data represents triplicated results, and the experiment was repeated 3 times. For the statistical analysis, a two‐way ANOVA test combined with Bonferroni's correction was used; Bars represent mean ± SEM. Levels of significance are as follows: ns, not significant; **, *P* < 0.01; ***, *P* < 0.001.

Various studies have shown that inhibition of MCE reduces adipogenesis efficiency [2, 35, 36, 37]. However, STIM2β KO 3T3‐L1 cells show increased differentiation efficiency with the reduced MCE rate. The intracellular Ca^2+^ signaling is also involved in the transcriptional regulation of various cellular processes. Therefore, we hypothesized that alteration in STIM2β could affect the transcription cascades to regulate adipogenesis. To test this hypothesis, we assessed the expression levels of C/EBPs and PPARγ2, which are known to regulate adipogenesis, in the early time point of differentiation (Supplementary Fig. S3A, Fig. 5A). qPCR analysis revealed that PPARγ2 mRNA expression was significantly increased in STIM2β KO L1 cells (Fig. 5A). To further investigate whether the increase in PPARγ2 mRNA expression leads to an increase in PPARγ2 protein expression, we analyzed the protein expression levels of PPARγ isoforms in differentiated WT and STIM2β KO 3T3‐L1 cell lines (Fig. 5B). The results showed increased expression of PPARγ2 in STIM2β KO L1 cell lines compared with the WT L1 cells (Fig. 5D). The expression of PPARγ1 was not significantly different between WT and STIM2β KO L1 cell lines (Fig. 5C). In addition, the expression of C/EBPα in WT and STIM2β KO cell lines did not differ significantly, confirming the PPARγ2‐specific increase in STIM2β‐deficient cells (Supplementary Fig. S3B,C).

PPARγ2 is the master regulator of adipogenesis, which is responsible for the expression of various adipocyte‐specific genes. To test whether increased PPARγ2 expression could affect its downstream target gene expression in STIM2β KO cell lines, we measured the mRNA expression of known PPARγ2 downstream target genes. RT‐PCR analysis of the mRNA expression of glucose transporter 4 (GLUT4) showed enhanced induction of the GLUT4 gene. In WT L1 cells, GLUT4 was induced on day 5 of differentiation, while it was induced on differentiation day 3 in STIM2β KO cell lines (Fig. 5E). qPCR analysis of GLUT4 levels confirmed significant enhancement of GLUT4 expression in STIM2β KO cell lines at differentiation day 3 (Fig. 5F). In addition, qPCR analysis of aP2 and LPL confirmed that PPARγ2 downstream target gene expression is accelerated in the absence of STIM2β (Fig. 5F). These results indicate that PPARγ2 expression and activity are significantly increased in STIM2β KO cell lines, which resulted in the enhanced adipogenesis efficiency of STIM2β KO 3T3‐L1 cells.

To further confirm the role of STIM2β in the regulation of PPARγ2 transcription, we examined whether PPARγ2 transcription is dependent on the [Ca^2+^]_i_ regulated by STIM2β. Treatment of the WT and STIM2β KO 3T3‐L1 cells with MDI during 12 h of differentiation revealed no significant increase in PPARγ2 mRNA in WT cells, wherein it was significantly enhanced in STIM2β KO #1 cells, as observed earlier. Moreover, SOCE inhibitor YM58483 did not affect the expression of PPARγ2 in WT 3T3‐L1 cells but significantly reduced it in STIM2β KO #1 cells, to a level similar to that of WT cells (Fig. 5G). The reduction of PPARγ2 mRNA expression levels after SOCE inhibitor treatment confirms that PPARγ2 transcription activation is dependent on the [Ca^2+^]_i_, which is regulated by STIM2β.

Intracellular Ca^2+^ and Ca^2+^‐responsive proteins could mediate transcriptional regulation. Therefore, we investigated which Ca^2+^‐responsive transcription factor could activate PPARγ2 promoter activity using the PPARγ2 promoter‐reporter system. The reporter system was generated by inserting about 1.4 kb promoter region of the mouse PPARγ2 gene into a vector encoding firefly luciferase (Fig. 5H). Various Ca^2+^‐responsive transcription factors, selected by PROMO binding site prediction, were co‐transfected with empty or PPARγ2 reporter plasmid into HEK 293 cells. Among the tested candidates, cAMP‐responsive element binding protein (CREB) significantly increased the PPARγ2 promoter activity by about 20‐fold (Fig. 5I). In addition, PROMO binding site prediction revealed that putative binding sites of NFATs and C/EBPs are adjacent to each other (Fig. 5J). According to previous studies, Nuclear factor of activated T cells (NFAT) and CCAAT/enhancer binding protein (C/EBP) cooperate to activate the PPARγ2 gene [38, 39]. PPARγ2 promoter assay results with C/EBPα and NFAT1 confirmed that PPARγ2 promoter activity was significantly increased when C/EBPα and NFAT1 were co‐expressed, which was not increased when either C/EBPα or NFAT1 was transfected, respectively (Fig. 5I). These results demonstrate that PPARγ2 transcriptional regulation is dependent on SOCE activity and its downstream Ca^2+^‐dependent transcription factors CREB and NFAT.

Taken together, STIM2β‐mediated [Ca^2+^]_i_ is involved in the transcription regulation of PPARγ2 during adipogenesis. Furthermore, our findings provide a piece of evidence that MCE is not a prerequisite step for adipocyte differentiation.

### Adipogenes is enhanced in STIM2β KO SVF cells

**Fig. 6:**
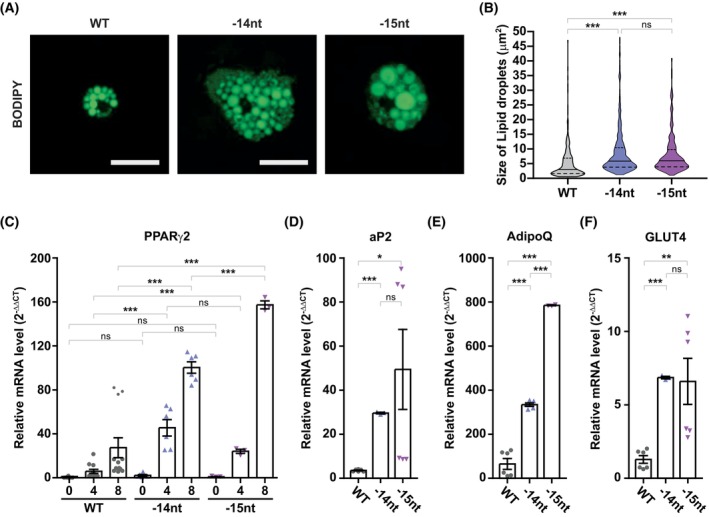
Alteration of STIM2β increases differentiation efficiency by enhancing peroxisome proliferator‐activated receptor gamma 2 (PPARγ2) expression in primary stromal vascular fraction (SVF) cells. (A) BODIPY staining showing the lipid accumulation in SVF cells that were induced to be differentiated by a hormonal cocktail for 8 days. This data is representative and the experiment was repeated 3 times. Scale bars indicate 50 μm; (B) Lipid droplet size analysis in (A). The size of a single lipid droplet was analyzed using the analyze particle function in imagej. This data represents the combined results from 3 repeated experiments. The one‐way ANOVA test combined with Bonferroni's Multiple Comparison Test was used to evaluate statistical significance. (C) *PPARG2* transcription levels in WT and STIM2β KO SVF cells were analyzed by qPCR at indicated time points after differentiation induction. This data represents triplicated results, and the experiment was repeated 3 times. Two‐way ANOVA with Bonferroni's correction was used for the statistical analysis. (D–F) Expression levels of PPARγ2 target genes fatty acid binding protein 4 (aP2) (D), adiponectin (AdipoQ) (E), and glucose transporter 4 (GLUT4) (F) were analyzed on differentiation day 4 by qPCR. This data represents triplicated results, and the experiment was repeated 3 times. The one‐way ANOVA combined with Dunnett's Multiple Comparison Test was used to evaluate statistical significance; Bars represent mean ± SEM. Levels of significance are as follows: ns, not significant; *, *P* < 0.05; **, *P* < 0.01; ***, *P* < 0.001.

To distinguish the kinetics of adipogenesis in primary STIM2β KO SVF cells, we examined the lipid accumulation by BODIPY staining in differentiated SVF cells. On differentiation day 8, we observed enlarged STIM2β KO SVF cells in comparison to WT SVF cells. This change in cell size implies that more lipid droplets were accumulated in STIM2β KO SVF cells (Fig. 6A). Furthermore, the size of each lipid droplet was increased by about 2‐fold in STIM2β KO SVF cells compared to that in WT cells (Fig. 6B), confirming that the differentiation efficiency is enhanced in the absence of STIM2β.

We further analyzed the mRNA expression of PPARγ2 in WT and STIM2β KO SVF cells during 8 days of differentiation. In agreement with the previous results observed in 3T3‐L1 cell lines, an increase in the PPARγ2 mRNA expression was observed in STIM2β KO SVF cells (Fig. 6C). Furthermore, the expression levels of the downstream target genes of PPARγ2, such as aP2, AdipoQ, and GLUT4, were significantly increased in STIM2β KO SVF cells compared to those in WT SVF cells (Fig. 6D–F). These data confirm that alteration of STIM2β in pre‐adipocytes enhances adipogenesis by increasing the expression of PPARγ2.

### STIM2β KO mice show hypertrophic adipose tissue development

Our data revealed that STIM2β regulates [Ca^2+^]_i_, which in turn regulates MCE and PPARγ2 transcription after adipogenic differentiation. Although there is a debate about the prerequisite of MCE for adipogenesis, MCE could benefit the cell population that undergoes the differentiation process. Hong *et al*. showed that embryonic pre‐adipocytes undergo active proliferation in embryonic white adipose tissue to expand the adipose‐lineage cell population [40].

**Fig. 7:**
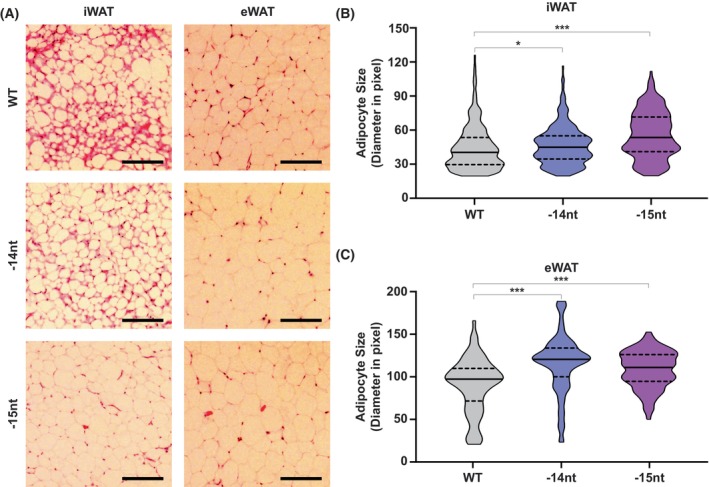
Hypertrophic white adipose tissue (WAT) development in STIM2β knockout (KO) mice. (A) H&E staining results of inguinal white adipose tissue (iWAT) and epididymal white adipose tissue (eWAT) of wild‐type (WT) and STIM2β KO mice. Images were acquired using EVOS. Scale bars indicate 100 μm. (B) Average adipocyte size analysis in iWAT of WT and STIM2β KO mice. To evaluate statistical significance, one‐way ANOVA combined with Dunnett's multiple comparison test was used. (C) Average adipocyte size analysis in eWAT of WT and STIM2β KO mice. One‐way ANOVA combined with Dunnett's multiple comparison test was used to evaluate the statistical significance. The histological analysis of WT and STIM2β KO mice was repeated 3 times; Bars represent mean ± SEM. Levels of significance are as follows: *, *P* < 0.05; ***, *P* < 0.001.

To delineate the effect of STIM2β KO on adipose tissue development, we analyzed the adipose tissues in WT (C57BL/6N) and STIM2β KO (C57BL/6N‐STIM2β^em1^) adult male mice. We analyzed the adipose tissue architecture of WT and STIM2β KO mice by hematoxylin/eosin staining (Fig. 7A). The average adipocyte size of iWAT was increased in STIM2β KO mice. The diameter of adipocytes in WT iWAT was smaller (<48 pixels in approximately 65% of the adipocytes) than that of those in STIM2β KO iWAT (>48 pixels in approximately 60% of adipocytes) (Fig. 7B). Hypertrophic adipocytes were also observed in eWAT in STIM2β KO mice. The average adipocyte size was significantly increased in STIM2β KO eWAT. In addition, STIM2β‐deficient eWAT showed a shifted distribution of adipocyte size, with most adipocytes presenting a diameter between 120 and 140 pixels, while most WT adipocytes present a diameter between 80 and 100 pixels (Fig. 7C).

The hypertrophic white adipose tissue development in STIM2β KO mice suggests that the proliferation rate of pre‐adipocytes during *in vivo* adipose tissue development was reduced, as observed in 3T3‐L1 cells *in vitro*. Collectively, our data confirm that STIM2β regulates the [Ca^2+^]_i_, which in turn regulates the cell proliferation rate and PPARγ2 transcription during adipogenesis.

## Discussions

The adipocyte differentiation process is influenced by various intracellular signaling pathways [41]. One of the key regulatory factors of intracellular signaling pathways, intracellular Ca^2+^, was shown to be related to adipocyte differentiation. However, the effect of intracellular Ca^2+^ on adipogenesis is controversial, showing opposite effects. Increased intracellular Ca^2+^ level ([Ca^2+^]_i_) by thapsigargin (TG) and A23187 prevents adipogenesis of 3T3‐L1 cells [33]. However, chelating intracellular Ca^2+^ in 3T3‐L1 pre‐adipocytes results in the inhibition of insulin‐activated Akt pathways, which are also involved in adipogenesis [34]. Ca^2+^ supplementation to porcine bone marrow mesenchymal stem cells was shown to activate the Akt signaling pathway and enhance adipogenesis [15]. Treatment of cyclosporine A (CsA), which is the calcineurin inhibitor, was shown to both promote [12] and inhibit [42] differentiation of the 3T3‐L1 cells. These conflicting roles of intracellular Ca^2+^ on adipogenesis imply the importance of regulation in [Ca^2+^]_i_ during adipogenesis. [Ca^2+^]_i_ modulation at different time points of adipogenesis could both inhibit and promote adipogenesis [13]. However, how [Ca^2+^]_i_ is changed, and the regulation mechanism of [Ca^2+^]_i_ during adipogenesis remains elusive. In the present study, we discovered that the expression of STIM2β, the inhibitor of SOCE, is upregulated during differentiation of 3T3‐L1 and SVF cells. In addition, the basal Ca^2+^ levels of 3T3‐L1 pre‐adipocytes and primary SVF cells decreased after differentiation as STIM2β expression increased. STIM2β KO confirmed that the change in [Ca^2+^]_i_ is mediated by STIM2β during adipogenesis.

SOCE regulates various cellular processes such as proliferation, transcription regulation, and apoptosis [22, 31]. MCE during differentiation induction is a unique process for adipocyte differentiation, and Ca^2+^‐related signaling cascades were shown to regulate the MCE during adipogenesis [33, 35]. Concordantly, our study shows that alteration of STIM2β‐mediated intracellular Ca^2+^ homeostasis inhibits MCE after differentiation induction. A previous study has shown that lanthanides promoted the MCE process in 3T3‐L1 pre‐adipocytes [43]. Together, these results suggest that STIM2β and SOCE are the potential regulators of MCE during adipogenesis. The MCE phase precedes the terminal differentiation, but its prerequisite for the terminal differentiation is under debate [2, 3, 4, 44]. Our results with STIM2β KO 3T3‐L1 pre‐adipocytes indicated that proliferation and differentiation are regulated independently since the differentiation efficiency is enhanced even though the MCE is inhibited.

The enhanced differentiation efficiency by STIM2β KO indicates that intracellular Ca^2+^ signaling and higher SOCE activity function as a positive regulator for the terminal differentiation process. The involvement of STIM2β‐mediated intracellular Ca^2+^ in the regulation of both MCE and transcription machinery for adipogenesis might contribute to the controversial effect of intracellular Ca^2+^ on adipogenesis, which provides difficulty in studying the role of Ca^2+^ in adipogenesis. However, it also highlights the importance of precise regulation of [Ca^2+^]_i_ for proper differentiation. Using STIM2β KO mice, we demonstrated that decreased MCE could result in the reduction of adipose tissue hyperplasia, with fewer mature adipocytes. However, whether these changes in adipose tissue development by alteration of STIM2β could affect the metabolism should be addressed for further studies.

We observed PPARγ2‐specific induction after differentiation in STIM2β KO L1 cells, which is mediated by Ca^2+^‐responsive transcription factors, CREB, and NFAT. The increase in PPARγ2 expression by STIM2β KO was also observed in primary SVF cells. However, we could not observe the increase in C/EBPα in STIM2β KO L1 cell lines, even after the increase of PPARγ2 expression. These findings are consistent with that of a previous study, which showed that C/EBPα expression was reduced in STIM1 over‐expressed 3T3‐L1 cells [16]. Collectively, these findings suggest the existence of unknown Ca^2+^‐related mechanisms to regulate the expression of C/EBPα.

In conclusion, our study provides evidence that SOCE is involved in the regulation of adipogenesis, and STIM2β is the unknown regulator of [Ca^2+^]_i_ during the differentiation process to regulate MCE and PPARγ2 transcription, which balances the cell proliferation and differentiation processes.

## Materials and methods

### 3T3‐L1 cell culture and adipocyte differentiation

3T3‐L1 pre‐adipocytes (RRID:CVCL_0123; ATCC, Manassas, VA, USA) were cultured in a growth medium of Dulbecco's modified Eagle's medium (DMEM) with high glucose (Gibco; Thermo Fischer Scientific, Inc., Waltham, MA, USA) supplemented with 10% (v/v) bovine serum (Gibco; Thermo Fischer Scientific, Inc.). The differentiation medium comprised DMEM supplemented with 10% (v/v) fetal bovine serum (FBS; Merck, Darmstadt, Germany). Cells were grown until 2 days post‐confluence (day 0) and then treated for 3 days with differentiation medium containing MDI (0.5 mm methyl‐isobutyl‐xanthine, 1 μm dexamethasone, and 10 μg·mL^−1^ insulin; all purchased from Sigma‐Aldrich, St. Louis, MO, USA) to induce adipocyte differentiation. Subsequently, the cells were retreated with the differentiation medium containing 10 μg·mL^−1^ insulin on day 3 and every 2 days thereafter. Cells were maintained and differentiated at 37 °C in a 5% CO_2_ incubator.

### Intracellular Ca^2+^ measurements

Ratio‐metric Ca^2+^ imaging was performed by dual excitation ratio images using 340 and 380 nm UV laser lines with an IDX81 microscope (Olympus Corp., Tokyo, Japan). Cells were loaded with 10 μm Fura‐2/AM (Invitrogen; Thermo Fisher Scientific, Inc., Waltham, MA, USA) in DMEM at 37 °C for 30 min. Images were acquired in 2 mm or 0 mm Ca^2+^ Ringer's solution. Cells were treated with 1 μm thapsigargin in 0 mm Ca^2+^ solution to induce ER‐store depletion for 5 min. After ER‐store depletion, a 2 mm Ca^2+^ solution was perfused to measure Ca^2+^ influx. Images were processed with Metamorph (Molecular Devices, San Jose, CA, USA) and analyzed with Igor Pro® software (Wavemetrics, Inc., Lake Oswego, OR, USA).

### Generation of STIM2β KO 3T3‐L1 cell lines

We used the CRISPR/Cas9 system to target mouse STIM2β as described earlier [27]. The guide RNA sequence for the mouse STIM2 exon 9 region (5′‐CCTGCAGGTTAGTAGTTACTAGA‐3′) was cloned into the pRGEN vector (pRGEN‐mSTIM2β) (ToolGen, Inc., South Korea). 3T3‐L1 cells were transfected with pRGEN‐mSTIM2β, pRGEN‐Cas9, and pRGEN‐reporter using Lipofectamine 2000 (Invitrogen; Thermo Fisher Scientific, Inc., Waltham, MA, USA), according to the manufacturer's instructions. 2 days post‐transfection, cells were selected with 1 mg·mL^−1^ hygromycin for 4 days to generate single‐cell colonies. After one week, colonies were isolated, and genomic deletion along with mRNA depletion was analyzed by Sanger sequencing and RT‐PCR assay.

### Oil red O staining

Cells were fixed with 4% paraformaldehyde solution (PFA; Electron Microscopy Sciences, Hatfield, PA, USA) and stained with the lipophilic dye Oil Red O (Sigma‐Aldrich, St. Louis, MO, USA) according to the manufacturer's instructions. Stained cells were visualized by bright field microscopy (EVOS XL Core, AMEX‐1000; Thermo Fischer Scientific, Inc., Waltham, MA, USA), and the total lipid accumulation was analyzed by measuring the absorbance of Oil Red O resolved in 100% isopropanol (IPA; Merck, Darmstadt, Germany) using a SPECTRA MAX190 plate reader (Molecular Devices, San Jose, CA, USA) and SoftMAX® Pro5 software.

### Animals

STIM2β KO mice (C57BL/6N‐stim2β^em1^) were generated by using the CRISPR/Cas9 system. Briefly, Cas9 mRNA and sgRNAs were microinjected into fertilized embryos of C57BL/6N mice. Mutations in STIM2 exon 9 were confirmed by Sanger sequencing analyses (Macrogen Ltd., Korea). Homozygous KO mice were maintained from a homozygous intercross and used for phenotypic analyses in parallel with age‐ and sex‐matched wild‐type (WT) littermates as a control group. All mice were genotyped 3 weeks after birth using PCR with specific primers (forward, 5′‐GTAGTCTCTTATTTCATGATCAAT‐3′ and reverse, 5′‐TATCAGCCAAGATACAGACATGTT‐3′), and animals were housed according to their gender after weaning. Mice were kept in an enriched environment under the standard conditions (22 ± 2 °C temperature, 40–60% humidity) with a 12/12 h light/dark cycle at the Specific‐Pathogen‐Free (SPF) facility. All experimental procedures were approved by the Ulan National Institute of Science and Technologies Institutional Animal Care and Use Committee (UNISTIACUC) (Approval no. UNISTIACUC‐21‐03).

### BODIPY staining

BODIPY 493/503 (Invitrogen; Thermo Fisher Scientific, Inc., Waltham, MA, USA) and the cells were stained following the manufacturer's instructions. Briefly, 10 mg BODIPY 493/503 was dissolved in 7.7 mL DMSO to make a 5 mm stock solution, which was stored at −20 °C before use. BODIPY staining solution (2 μm BODIPY, 1:25 000 diluted stock solution in PBS) was applied to the differentiated cells and incubated in a 37 °C incubator for 15 min. Cells were then washed with PBS to remove excess, unbound BODIPY and then fixed with 4% PFA for 10 min. After fixation, cells were incubated with Hoechst 33342 (Thermo Fischer Scientific, Inc.) to stain the nucleus for 20 min and analyzed by an epi‐fluorescent microscope (IX83; Olympus Corp., Tokyo, Japan). The number, size, and intensity of lipid droplets were measured using the Analyze Particle function of imagej software.

### Mouse primary cell culture and differentiation

Dissected inguinal white adipose tissue from male mice at the age of 9–10 weeks was chopped with scissors and incubated in the digestion medium containing 100 mm HEPES buffer (pH7.4), 120 mm NaCl, 50 mm KCl, 5 mm d‐Glucose, 1 mm CaCl_2_, 1.5% BSA, and 10 mg·mL^−1^ Type I collagenase (Worthington Biochemical Corp., Lakewood, NJ, USA) for 1 h at 37 °C. The resulting suspension was filtered through 70 μm nylon strainers (BD Falcon; Corning, NY, USA) to remove undigested parts, followed by centrifugation (500 ***g*** for 10 min). After several washes, the stromal vascular fraction (SVF) pellet was re‐suspended in the culture medium comprising DMEM/F‐12 (Gibco; Thermo Fischer Scientific, Inc.), 10% (v/v) FBS, 100 units·mL^−1^ penicillin, and 100 μg·mL^−1^ streptomycin (Pen/Strep; Gibco; Thermo Fischer Scientific, Inc.) at 37 °C in a 5% CO_2_ incubator. Differentiation was induced by treating post‐2‐day confluent cells with the differentiation induction medium containing 0.5 mm isobutyl methylxanthine, 5 μm dexamethasone, and 1 μg·mL^−1^ insulin in the growth medium. After 3 days, the adipogenic differentiation induction medium was changed to the maintenance medium containing 1 μg·mL^−1^ insulin.

### Mouse white adipose tissue histology analysis

The white adipose tissues of 8‐ to 10‐week‐old male mice were fixed in 4% PFA, embedded in paraffin, and sectioned. Sections were subjected to hematoxylin and eosin (H&E) staining. Bright Field images were taken using an EVOS XL Core microscope. The number and size of adipocytes were measured using the imagej software with the Adiposoft plugin.

### Protein expression analysis using immunoblotting

Cells were lysed in lysis buffer containing 0.1% sodium dodecyl‐sulfate (SDS), and samples were centrifuged. Supernatants were immediately boiled with 1× SDS loading buffer at 95 °C for 10 min. Protein extracts prepared from cells harvested at the indicated times post‐differentiation were resolved using Sodium dodecyl‐sulfate polyacrylamide gel electrophoresis (SDS/PAGE) and protein transfer was transferred onto a PVDF membrane (Millipore, Burlington, MA, USA). Membranes were blocked for 2 h with 7% skim milk in TBS‐T buffer (TBS, 0.1% Tween 20), and were incubated with primary antibodies overnight at 4 °C. Immunoblot analysis was performed using the ECL western blotting detection system (Bio‐Rad, Hercules, CA, USA). For the quantification of PPARγ isoforms, protein transfer was transferred onto a Nitrocellulose membrane (Cytiva, Marlborough, MA, USA), and fluorescent secondary antibodies were used in the Odyssey Clx Infrared fluorescent western blotting system (Li‐Cor Biosciences, Lincoln, NE, USA). The following antibodies were used: anti‐PPARγ (Cat# 2443S; Cell Signaling Technology, Danvers, MA, USA), anti‐C/EBPα (Cat# SC‐365318; Santa Cruz Biotechnology, Dallas, TX, USA), and anti‐β‐actin (Cat# SC‐47778); secondary antibodies conjugated with HRP (Goat anti‐mouse IgG and Goat anti‐rabbit IgG; Millipore, Burlington, MA, USA); fluorescent secondary antibodies (IRDye® 800CW Goat anti‐Rabbit and IRDye® 680RD Goat anti‐Mouse; Li‐Cor Biosciences, Lincoln, NE, USA).

### RNA extraction and quantitative analysis

Total RNA was extracted from cells using phenol‐chloroform‐based methods and quantified using Nanodrop 2000c (Thermo Fischer Scientific, Inc., Waltham, MA, USA). Approximately 1.1 μg RNA was converted to cDNA using ReveTra ACE™‐α cDNA synthesis kit (Toyobo, Osaka, Japan) according to the manufacturer's instructions. cDNA was then analyzed by either RT‐PCR using Thermal cycler C1000 (Bio‐Rad, Hercules, CA, USA) or quantitative real‐time PCR analysis using SYBR green (Toyobo, Osaka, Japan) with Light Cycler 480 II instrument (Roche, Basel, Switzerland). The primer sequences used for RT‐PCR analysis are shown in Table 1, and those used for qPCR analysis are shown in Table 2.

**Table 1:** Primer sequence information used in RT‐PCR.

Target gene		Sequence (5′–3′)
STIM2α	Forward	GCTAGCCATCGCTAAGGACGAGGCAG
	Reverse	AGCTATCTGAAAGCCACAGATCTTCTC
STIM2β	Forward	TCGCTGCCTCCTATCTCCTGCAGG
	Reverse	ATGGCACGATGCTGCGACGA
PPARγ2	Forward	TCTGGGAGATTCTCCTGTTGA
	Reverse	GGTGGGCCAGAATGGCATCT
aP2	Forward	GAATTCGATGAAATCACCGCA
	Reverse	CTCTTTATTGTGGTCGACTTTCCA
AdipoQ	Forward	CTACTGTTGCAAGCTCTCC
	Reverse	CTTCACATCTTTCATGTACACC
GLUT4	Forward	GTAACTTCATTGTCGGCATGG
	Reverse	AGCTGAGATCTGGTCAAACG
36B4	Forward	AATCTCCAGAGGCACCATTG
	Reverse	CCGATCTGCAGACACACACT
Actin	Forward	GATCTGGCACCACACCTTCT
	Reverse	GGGGTGTTGAAGGTCTCAAA

**Table 2:** Primer sequence information used in qPCR.

Target gene		Sequence (5′–3′)
STIM2α	Forward	GCTAGCCATCGCTAAGGACGAGGCAG
	Reverse	AGCTATCTGAAAGCCACAGATCTTCTC
STIM2β	Forward	TCGCTGCCTCCTATCTCCTGCAGG
	Reverse	GCACGTTGCACACAGCTCCTCCCTGGA
PPARγ2	Forward	ATGCACTGCCTATGAGCACT
	Reverse	CAACTGTGGTAAAGGGCTTG
aP2	Forward	GAAATCACCGCAGACGACAGG
	Reverse	GCTTGTCACCATCTCGTTTTCTC
AdipoQ	Forward	GTCAGTGGATCTGACGACACCAA
	Reverse	ATGCCTGCCATCCAACCTG
GLUT4	Forward	GTAACTTCATTGTCGGCATGG
	Reverse	AGCTGAGATCTGGTCAAACG
C/EBPβ	Forward	GGTTTCGGGACTTGATGCA
	Reverse	CAACAACCCCGCAGGAAC
C/EBPα	Forward	CGCAAGAGCCGAGATAAAGC
	Reverse	CAGTTCACGGCTCAGCTGTTC
36B4	Forward	AATCTCCAGAGGCACCATTG
	Reverse	CCGATCTGCAGACACACACT

### Growth rate calculation

The growth rate of 3T3‐L1 cells was calculated by using the following equation:

$$\text{Grwoth rate}=\frac{\ln\left(\frac{N_{t}}{N_{0}}\right)}{t}$$

where *N*_*t*_ is the cell number at the indicated time and *N*_0_ is the initial cell number.

### Cell cycle analysis using flow cytometry

3T3‐L1 cell lines were induced to be differentiated as described above. Cell monolayers at the desired time point after differentiation induction were washed with PBS, trypsinized, and detached cells were diluted in ice‐cold PBS. Cells were gently pelleted by centrifugation (300 ***g***, 5 min). After removing PBS, cells were fixed and permeabilized with a drop‐wise addition of 90% ice‐cold ethanol while vortexing. Fixed cells were washed with PBS and incubated in the dark for 30 min with propidium iodide (PI) staining solution containing 50 μg·mL^−1^ PI and 100 μg·mL^−1^ RNase A (both were purchased from Invitrogen; Thermo Fisher Scientific, Inc., Waltham, MA, USA) in PBS. DNA fluorescence was measured with a FACS Calibur Flow Cytometer (BD Biosciences, San Jose, CA, USA) equipped with a 488‐nm argon laser. Width (FL2W) and area (FL2A) of PI fluorescence were recorded for at least 10 000 counts. The percentage of cells in each phase of the cell cycle was analyzed using flowjo software.

### PPARγ2 promoter assay

PPARγ2 promoter region of 3T3‐L1 genomic DNA was amplified by PCR and subcloned into a pGL4.10 vector using NdeI and BglII (New England Biolabs, Ipswich, MA, USA). PPARγ2 promoter reporter was transfected to HEK293 cells (RRID:CVCL_0045) with renilla vector (pRLTK) at a ratio of 4 : 1. Luciferase activity was measured using a GLOMAX® luminometer (Promega, Madison, WI, USA) and normalized by renilla activity.

All cell lines were tested for mycoplasma contamination on a monthly basis, and only mycoplasma‐free cells were used in this study.

## Conflict of interest

The authors declare no conflict of interest.

## Author contributions

SJJ performed experiments, analyzed and visualized data, and wrote the manuscript. B‐WS and S‐UK generated knockout mouse lines. CYP supervised the study.

## Peer review

The peer review history for this article is available at https://www.webofscience.com/api/gateway/wos/peer‐review/10.1111/febs.70118.

## Supporting information


**Fig. S1.** Generation of STIM2β knockout (KO) 3T3‐L1 cell lines.
**Fig. S2.** Effect of Store‐operated Ca^2+^ entry (SOCE) inhibitors in 3 T3‐L1 pre‐adipocyte during differentiation.
**Fig. S3.** Analysis of mRNA and protein expression level of C/EBPα.

## Data Availability

The data that support the findings of this study are available from the corresponding author (cypark@unist.ac.kr) upon reasonable request.
