# Supplementary material for: STIM2β is a Ca2+ signaling modulator for the regulation of mitotic clonal expansion and PPARG2 transcription in adipogenesis
Source: FEBS J. 2025 May 9;292(15):4018–38. doi: 10.1111/febs.70118 (PMC12326919; doi:10.1111/febs.70118)
Supplement: Supplementary file 1 — Fig. S1. Generation of STIM2β knockout (KO) 3T3‐L1 cell lines. Fig. S2. Effect of Store‐operated Ca2+ entry (SOCE) inhibitors in 3 T3‐L1 pre‐adipocyte during differentiation. Fig. S3. Analysis of mRNA and protein expression level of C/EBPα. [file FEBS-292-4018-s001.pdf]

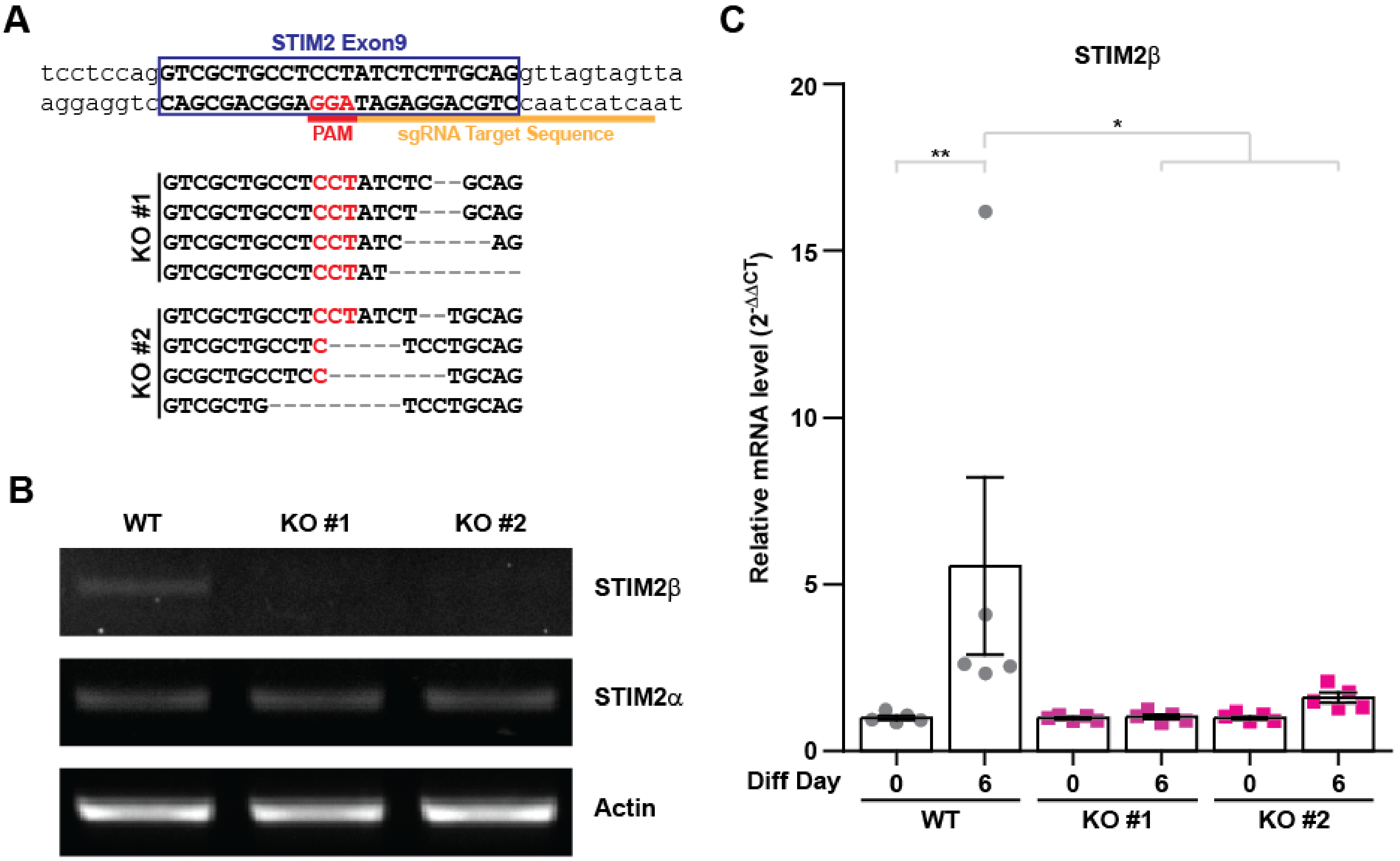


**Supplementary Figure S1. Generation of STIM2β knockout (KO) 3T3-L1 cell lines.**

(A) STIM2 exon9 sequence information of STIM2β KO 3T3-L1 cell lines.

(B) Confirmation of KO efficiency using RT-PCR analysis.

(C) qPCR analysis of STIM2β mRNA expression after differentiation in WT and STIM2β KO 3T3-L1 cell lines. 5 different sets of cDNAs were used. For the statistical analysis, the one-way ANOVA combined with Bonferroni's Multiple Comparison test was used. Bars represent mean±SEM; Levels of significance are as follows: *, $p<0.05$; **, $p<0.01$;

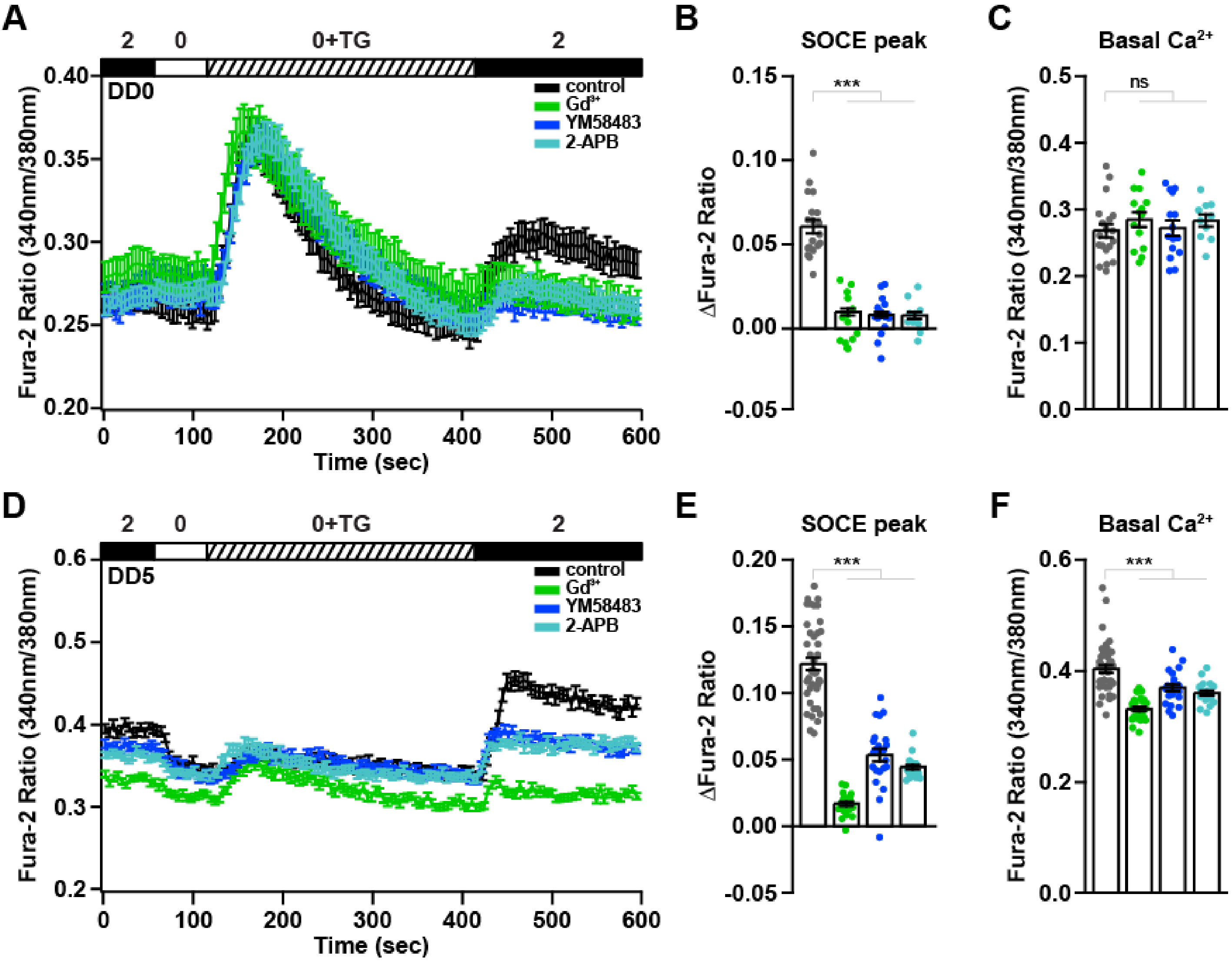


**Supplementary Figure S2. Effect of SOCE inhibitors in 3T3-L1 preadipocyte during differentiation.**

(A) $[Ca^{2+}]_i$ analysis of 3T3-L1 cells at differentiation day 0 (DD0) with Orai1 blockers. $[Ca^{2+}]_i$ were expressed as ratios of 340:380nm Fura-2 fluorescence signals. (control, n = 42; $Gd^{3+}$, n = 26; YM68483, n = 23; 2-APB, n = 20); (B) Bar graph presents the SOCE peak after 2mM $Ca^{2+}$ add-back in (A). The SOCE peak was calculated by subtracting the minimum Fura-2 ratio from the maximum Fura-2 ratio. These ratios were obtained by averaging the values from three imaging points preceding and following the respective maximum and minimum points; (C) Bar graph presents the basal $Ca^{2+}$ levels of cells in (A). Basal $Ca^{2+}$ level was calculated by averaging Fura-2 ratios of 340:380nm fluorescence signals from 30 to 60 seconds; (D) $[Ca^{2+}]_i$ analysis of 3T3-L1 cells at differentiation day 5 (DD5) with Orai1 blockers. $[Ca^{2+}]_i$ were expressed as ratios of 340:380nm Fura-2 fluorescence signals. (WT, n = 62; -14nt, n = 28; -15nt, n=30); (E) Bar graph presents the SOCE peak after 2mM $Ca^{2+}$ add-back in (D). The SOCE peak was calculated by subtracting the minimum Fura-2 ratio from the maximum Fura-2 ratio. These ratios were obtained by averaging the values from three imaging points preceding and following the respective maximum and minimum points; (F) Bar graph presents the basal $Ca^{2+}$ levels of cells in (D). Basal $Ca^{2+}$ level was calculated by averaging Fura-2 ratios of 340:380nm fluorescence signals from 30 to 60 seconds; One-way ANOVA test combined with Dunnett's Multiple Comparison test was used to evaluate statistical significance; Bars represent mean ± SEM; Levels of significance are as follows: ns, non-significant; ***, $p < 0.001$;

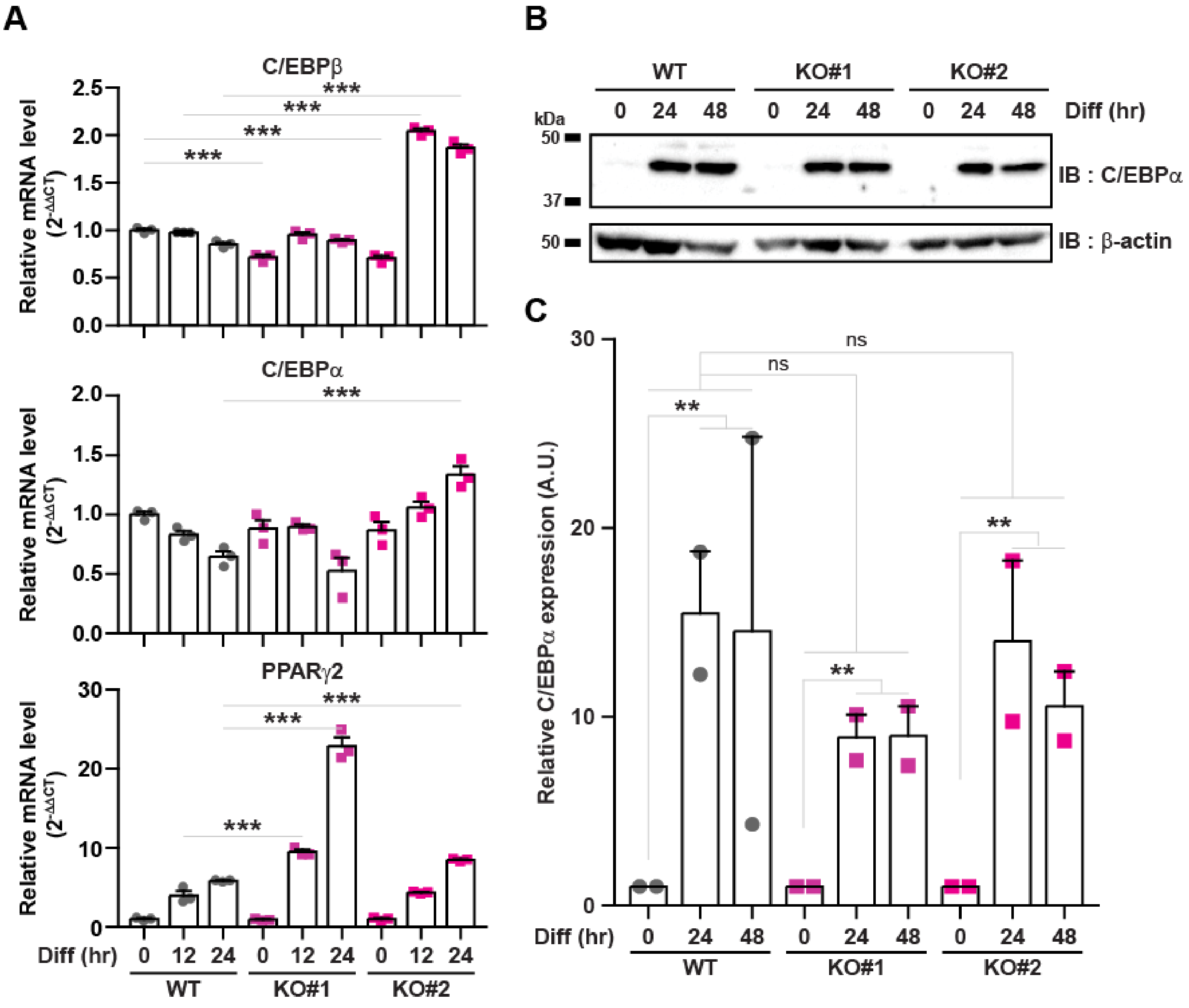


**Supplementary Figure S3. Analysis of mRNA and protein expression level of C/EBPα.**

(A) mRNA expression analysis of adipogenic transcription factors C/EBPβ, C/EBPα, and PPARγ2 at early-time points of differentiation using qPCR;

(B) C/EBPα protein expression analysis of WT and STIM2β KO 3T3-L1 cell lines after differentiation by western blotting;

(C) C/EBPα expression level was analyzed by measuring the band intensity of C/EBPα to β-actin at each time point. Two independent blots were used. For the statistical analysis, two-way ANOVA test with Bonferroni's correction was used; Bars represent mean ± SEM; Levels of significance are as follows: ns, non-significant; **, $p < 0.01$; ***, $p < 0.001$;
